# Multi-Omics Landscape of DNA Methylation Regulates Browning in “Fuji” Apple

**DOI:** 10.3389/fnut.2021.800489

**Published:** 2022-02-07

**Authors:** Lihua Wang, Tiantian Tang, Wenjun Wang, Jie Zhang, Zhidong Wang, Fengzhong Wang

**Affiliations:** Key Laboratory of Agro-Products Quality and Safety Control in Storage and Transport Process, Ministry of Agriculture and Rural Affairs, Institute of Food Science and Technology, Chinese Academy of Agricultural Sciences, Beijing, China

**Keywords:** apple, browning, DNA methylation, multi-omics landscape, *NCA1*

## Abstract

Browning seriously affects the quality of fresh-cut fruits, and its mechanism was thought to be polyphenol oxidase (PPO) in the past. A way of non-different PPO browning was found in our previous studies. However, the landscape of this browning way is still unclear in “Fuji” apples. Multi-omics (methylomics, transcriptomics, and proteomics) methods were performed to the global profiles of DNA methylation and gene and protein expression. We employed two natural bud mutation varieties of apple as materials and found a positive correlation between browning index (BI) and methylation (5mC%, *MdCMT3*, and *MdCMT3c*) and a negative correlation between BI and demethylation (*MdROS1* and *MdDME*). DNA methylation inhibitor 5-azacytidine can delay apple browning. Further analysis showed that methylated-*NCA1* and OMT1 increased significantly in apple browning. Methylated-*NCA1* might inhibit *NCA1* gene expression and resulted in the decline of catalase activity, thereafter significantly increased apple browning. These findings insight into a new pathway and landscape that DNA hypermethylation significantly accelerated the browning in “Fuji” apple.

## Highlights

- Apple browning was closely related to DNA methylation, and DNA methylation level significantly increased with the increase of browning.
- Multi-omics (methylomics, transcriptomics, and proteomics) landscape is displayed in apple browning.

## Introduction

Apple is one of the favorite fruits of mankind and originated in China (1, 2). Apples have high nutritional value and help to improve human health (3). In recent years, with the increasing demand for fresh, healthy, convenient, and nutritious fruits by consumers, fresh-cut fruits have gradually become a new aspect with the fastest development in the food industry (4). However, the storage problem of fresh-cut fruits is very serious mainly due to excessive tissue browning. Browning affects the appearance, flavor, and nutritional value which causes significant economic losses (5). Therefore, it is of great value for improving the quality of fruit that uncover the reasons and mechanisms of apple browning.

About 20 years ago, the higher activity of polyphenol oxidase (PPO) was found that is a main reason of apple browning (6–9). In recent years, antibrowning apple (Arctic^®^ Apples) was developed by reducing the expression of PPO gene (10). However, fruit browning is a complex process which is affected a variety of external (temperature, light, etc.) and internal factors (genes, proteins, and metabolites including flavonoids and polyphenols) (8, 9). In the past three years, our team found that bud mutation apples have a significant difference at the level of browning, but there is no difference in the activity of PPO (11). This browning difference may be caused by epigenetic factors such as DNA methylation. Moreover, treatment with the DNA methylation inhibitor 5-azacytidine (5′-Aza) was found to induce accumulation of anthocyanin compounds in peach flesh, demonstrating a functional impact of DNA demethylation on polyphenol levels in peach flesh (12). DNA methylation regulates gene expression and affects plant growth and development (13–17), and also fruit maturation (18–24). The level of DNA methylation was gradually increased during the fruit maturation which indicated DNA hypermethylation was crucial for the maturation in sweet orange (25). Research on DNA methylation has moved from model plants to more crops (26). The multi-omics provides a comprehensive approach to understand biological processes that integrate DNA methylation with other omics, including transcriptomics, proteomics, and metabolomics (27, 28). The embryonic development of cotton fiber (29), leaf senescence (30), and tomato pathogen response (31) was studied by means of multi-omics. In apple, the results of multi-omics studies are mainly in quality control (32), flower bud development (33), and DNA methylation on the accumulation of soluble sugars and organic acids (34). Therefore, multi-omics has played an increasingly important role in the study of plants and apples.

In this study, we analyzed a positive correlation between browning index (BI) and methylation (5mC%, *MdCMT3*, and *MdCMT3c*) and a negative correlation between BI and demethylation (*MdROS1* and *MdDME*). Moreover, multi-omics that includes methylomics, transcriptomics, and proteomics analysis has been showed that methylated-*NCA1* and O-methyltransferase 1 (OMT1) significantly increased in apple browning. We proposed that methylated-*NCA1* might inhibit to *NCA1* and resulted in the decline of catalase (CAT) activity, thereafter significantly increasing apple browning. This work has provided a new point and evidence for understanding the process of browning.

## Materials and Methods

### Apple Collection

Fruits of two “Fuji” apple types (*Malus domestica* Borkh.), “flushed-skin color pattern” (abbreviated as P-type) and “striped-skin color pattern” (abbreviated as T-type), were hand-harvested from the orchard located in Beijing of China on October, 2019. Fruits were selected for uniformity in maturity stage, size, and shape and also the absence of mechanical damage and disease. Freshly harvested apples were transported immediately to the Institute of Food Science and Technology, Chinese Academy of Agricultural Sciences (Beijing, China), and stored in dark at 4°C until for use.

### Sample Treatment and Experiment Design

Each apple was cut into eight pieces after peeling and then were put in a fresh-keeping box in a refrigerator for 2 h. The T-type apples were cut into 1-cm^3^ pieces and immersed in 50 mM 5′-Aza solution (5′-Aza, dissolved in 10% DMSO solution), whereas the control sample (CK) was immersed in 10% DMSO solution. The photos were taken and the BI value was measured after 2 h.

### The Determination of BI

The BI value of apples was measured using a DigiEye Electronic Eye (DigiEye Digital Imaging System, Verivide, USA) according to the CIEL^*^a^*^b^*^ within 5 h of storage. At the first 3 h, we measured every half an hour and measured every hour after 3 h. Two apple types measured immediately after cutting were as 0-h samples and measured after cutting after 2 h were as 2-h samples. Then, the samples were labeled as P0, T0, P2, and T2, respectively. After the BI measurement was completed, apple samples of P0, T0, P2, and T2 were cut into small pieces, placed in liquid nitrogen for quick freezing, and stored in a refrigerator at−80°C. BI was calculated according to Eq. (1) (35).


(1) $$\begin{array}{r} \text{BI }=\;\frac{100\;\times \left[\left[\frac{\left[a^{2}+\left(1.75\times L\right)\right]}{\left[\left(5.645\times L\right)+a^{2}-\left(3.012\times b\right)\right]}\right]-0.31\right]}{0.172} \end{array}$$

### The Determination of CAT Activity

Apples were ground with 800 μL extraction buffer (10 mM Tris-HCl, 150 mM NaCl, 2 mM EDTA, and 0.5% polyvinylpyrrolidone PvP-30), centrifuged at 10,000 g for 10 min at 4°C, and the supernatant liquid was used to determine the CAT activity. The CAT activity determination kit (Beyotime Biotechnology Co., Ltd., Shanghai, China) was used to test the CAT activity according to the instructions and the previous study (36). The supernatant liquid (40 μL) was mixed with 250 mM H_2_O_2_ solution, reacted at 25°C for 4 min, and CAT reaction stop solution was added to terminate the reaction. The mixture was added into the working color solution, incubated at 25°C for at least 15 min, and measured the absorbance at 520 nm. CAT activity was expressed in units/g. One unit of CAT activity was defined as the ability to catalyze the decomposition of 1 μmoL of H_2_O_2_ per minute.

### DNA Methylation Detection by LC-MS/MS

According to Friso et al. (37) method, 1 μg of DNA was denatured by heating at 100°C for 3 min and subsequently chilled in refrigerator at 4°C for 10 min. One-tenth volume of 0.1 M ammonium acetate (pH 7.5) and 2 units of DNase I (NEB, USA) were added. The mixture was then incubated at 37°C for 3 h. Two units of alkaline phosphatase (NEB, USA) were subsequently added. The incubation was continued for an additional 3 h at 37°C. Thereafter, the mixture was incubated overnight at 37°C with 40 units of exonuclease I (Takara Biomedical Technology Co., Ltd., Beijing, China). The complete lysis mixture was placed in a refrigerator at 4°C for LC-MS/MS detection.

### Bis-Sequence for DNA Methylomics

DNA extraction was performed according to CTAB method (38–40). Bis-sequence was performed by Shanghai BIOZERON Co., Ltd. Before bisulfite treatment, 25 ng lambda-DNA was added to the 5 μg genomic DNA. Then, the mixed DNA was fragmented with a Sonicator (Sonics &Materials) to 300 bp. Differentially methylated regions (DMRs) were searched using a 200 bp sliding-window with 50 bp as step-size by R methyl Kit package. Windows with the false discovery rate (FDR) less than 0.05 and an over 2-fold change in the methylation level were retained for DMR.

### Transcriptome Analysis by RNA-Sequencing

Total RNA was extracted from 0.4 g frozen flesh samples using a Quick RNA Isolation Kit (Huayueyang Biotech Co., Ltd., Beijing, China) according to the manufacturer's instructions. The concentration and integrity of RNA samples were determined and assessed by NanoDrop One spectrophotometer (Thermo Fisher Scientific Inc., Waltham, MA, USA) and 1% (w/v) agarose gel. RNA-seq transcriptome libraries were prepared following TruSeq^TM^ RNA sample preparation Kit from Illumina (San Diego, CA, USA), using 1 μg of total RNA (41). The differentially expressed genes (DEGs) between two samples were selected using the following criteria: the logarithmic of fold change was greater than 2 and the FDR should be less than 0.05. To understand the functions of the DEGs, GO functional enrichment and KEGG pathway analysis were carried out by Goatools (https://github.com/tanghaibao/Goatools) and KOBAS (http://kobas.cbi.pku.edu.cn/home.do). DEGs were significantly enriched in GO terms and metabolic pathways when their Bonferroni corrected *p*-value was less than 0.05.

### Proteomics Analysis Using Label-Free Nano-LC-MS/MS

The experiments of proteomics were performed by Beijing ZhengDa Health Biomedical Technology Co., Ltd. The protein in the sample was extracted by sonication using denaturant and was cut into peptides by trypsin. Then, the sample was desalted using a C18 reversed-phase chromatography column. Data acquisition was based on liquid chromatography–tandem mass spectrometry. Orbitrap Fusion Lumos coupled Easy-nLC 1200 liquid chromatography system (Thermo Fisher Scientific, USA) was used to separate peptides. The raw data obtained by mass spectrometry were analyzed with the Proteome Discoverer 2.2 (Thermo Fisher Scientific, USA) using the integrated SEQUEST (42).

### Quantitative Real-Time Polymerase Chain Reaction (qPCR) and Gene Expression Analysis

EF-1α serves as reference gene from the previous studies (43–45) and methylated genes are according to the previous study (46). The 10 pair primers of targeted genes were designed by Primer Premier 6.0 software (PREMIER Biosoft, Palo Alto, CA, USA) and shown in Supplementary Table S2. Primers were synthesized by Beijing Tsingke Biotechnology Co., Ltd (Beijing, China).

Total RNA was isolated using 0.4 g frozen flesh samples using the Quick RNA isolation Kit (Huayueyang Biotech Co., Ltd., Beijing, China) according to the manufacturer's protocol. The concentration of RNA samples was determined by NanoDrop One spectrophotometer (Thermo Fisher Scientific Inc., Waltham, MA, USA), and integrity was assessed on 1% (w/v) agarose gel. The RNA samples with an A260/A280 ratio of 1.8–2.2 were used for qPCR. RNA reverse-transcription was performed after concentration normalization used QuantScript RT Kit (Tiangen Biotech Co., Ltd., Beijing, China).

The qPCR was performed on Bio-Rad CFX96 system (Bio-Rad Laboratories, Hercules, CA, USA). The total reaction volume of 20 μL contained 4 μL of diluted cDNA template, 0.8 μL of each primer (forward and reverse), 10 μL of SYBR Green fluorescent dyes, and 4.4 μL of ddH_2_O. The qPCR amplification procedure was set as 95°C for 3 min, 40 cycles of 95°C for 10 s, and 60°C for 30 s. One additional step was set at temperature 65–95°C with 0.5°C increase per second after the last cycle for melt curve analysis. All qPCR was completed with three biological replications, and the means of three replications were used as final quantification values. The 2^−ΔΔCT^ method was used for gene relative expression levels.

### Data Analysis and Drawing

Each sample has 3 replicates for DNA methylation, transcriptome, and proteomic experiments, 6 replicates for CAT activity and qPCR experiments, and 12 replicates for BI experiments. Graphing and data analysis were performed using GraphPad Prism 8.0. Pairwise comparisons were performed using *t*-test, and multiple comparisons were performed using ANOVA test. *p-Value* < 0.05 indicated significant differences. Statistical analysis data are presented as means ± standard error of the mean (SEM). The principal component analysis (PCA), correlation analysis, and Circos were performed in R language. To understand the functions of the DEGs and differentially expressed proteins, KEGG pathway analysis was carried out by KOBAS (http://kobas.cbi.pku.edu.cn/) (47).

## Results

### A Strong Positive Correlation Between DNA Methylation and Browning

**Figure 1:**
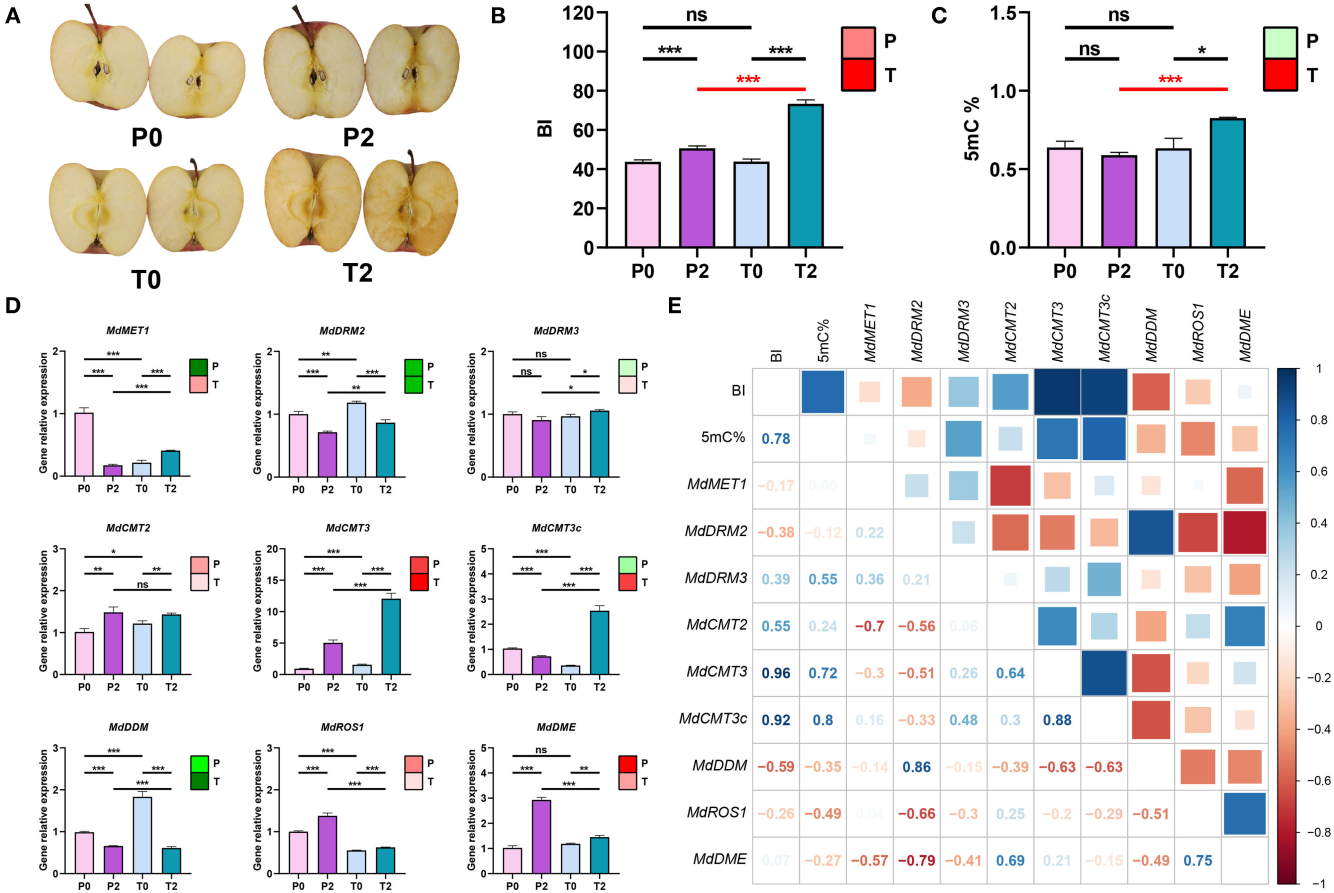
Relationship between browning and methylation in “Fuji” apples. **(A)** Photos of browning status of P-type and T-type “Fuji” apples at 0 and 2 h after cutting. P0, P-type apples at 0 h; P2, P-type apples at 2 h; T0, T-type apples at 0 h; T2, T-type apples at 2 h. **(B)** BI values. Note that for the color block in the upper right-hand corner, an increasing trend is indicated in red whereas a decreasing trend is indicated in green, the extent of a change is indicated by the color intensity [Nota Bene: the same coloring system is used **(C,D)**]. **(C)** 5 mC% of the genome in apples. The color block in the upper right-hand corner indicates an increase in red and a decrease in green. **(D)** Expression of known DNA methylation-related genes in apple. The nine genes shown here are *MdMET1, MdDRM2, MdDRM3, MdCMT2, MdCMT3, MdCMT3c, MdDDM, MdROS1*, and *MdDME*. The color block in the upper right-hand corner indicates an increase in red and a decrease in green. **(E)** Correlation matrix for BI and methylation-related data. Pearson's correlation analysis was performed between BI values, the 5 mC%, and the expression levels of the 9 genes in **(D)**. In the figures, ns indicates no significance, *p < 0.05, **p < 0.01, ***p < 0.001.

P-type and *T*-type apples are natural mutants from the bud mutation variety of “Fuji”. These two types of apples belong to the same variety (the same genetic background) and have the same growing environment (in the same orchard). However, the browning has significantly different and increased at 2 h time point after cutting in two types of “Fuji” apples (Figure 1A). For the 2 h time point, BI of the T-type apples were significantly higher than the P-type apples (*p* < 0.001) (Figure 1B). Pursuing the speculation that differential DNA methylation may impact apple browning, we used LC-MS/MS to determine 5mC% from four groups of apple samples (P0, P2, T0, and T2). For both P-type and T-type apples, there was a significant increase in the 5 mC% between the 0 and 2 h time points. Further, whereas there was no difference in the 5 mC% between the P-type and T-type apples at the 0 h time point, the 5 mC% was significantly increased in the T-type apples at the 2 h time point (*p* < 0.05) (Figure 1C).

These differential methylation results motivated us to use qPCR to assess the expression levels of nine genes with known DNA methylation-related function genes including *MdMET1, MdDRM2, MdDRM3, MdCMT2, MdCMT3, MdCMT3c, MdDDM, MdROS1*, and *MdDME* in the P-type and T-type apples (Figure 1D). *MdCMT3* and *MdCMT3c* encode a DNA methyltransferase that was significantly increased and higher in T-type apples (*p* < 0.001). But *MdCMT3c* was a significant decline in P-type apples. *MdROS1* and *MdDME* that encode demethylated genes have significantly increased and are higher in P-type apples.

We used a Pearson's correlation analysis to assess potential relationships among these data and found that BI is strongly positively correlated with 5 mC%, *MdCMT3*, and *MdCMT3c* and negatively correlated with *MdROS1* and *MdDME*. In addition, 5mC% is negatively correlated with *MdROS1* (Figure 1E). To further confirm the relationship between methylation and browning, T-type apples were treated with 5′-Aza (a DNA methyltransferase inhibitor) and found that the BI was significantly decreased as shown in Supplementary Figure S1.

Collectively, these results indicate that the extent of DNA methylation increased during the first 2 h of apple browning and the higher the expression of methylation-related genes. The higher the expression of demethylation-related genes, the slower the browning. The results revealed that browning was positively and strongly correlated with the increase of methylation.

### The Profiles and Types of Methylation by Methylomics

**Figure 2:**
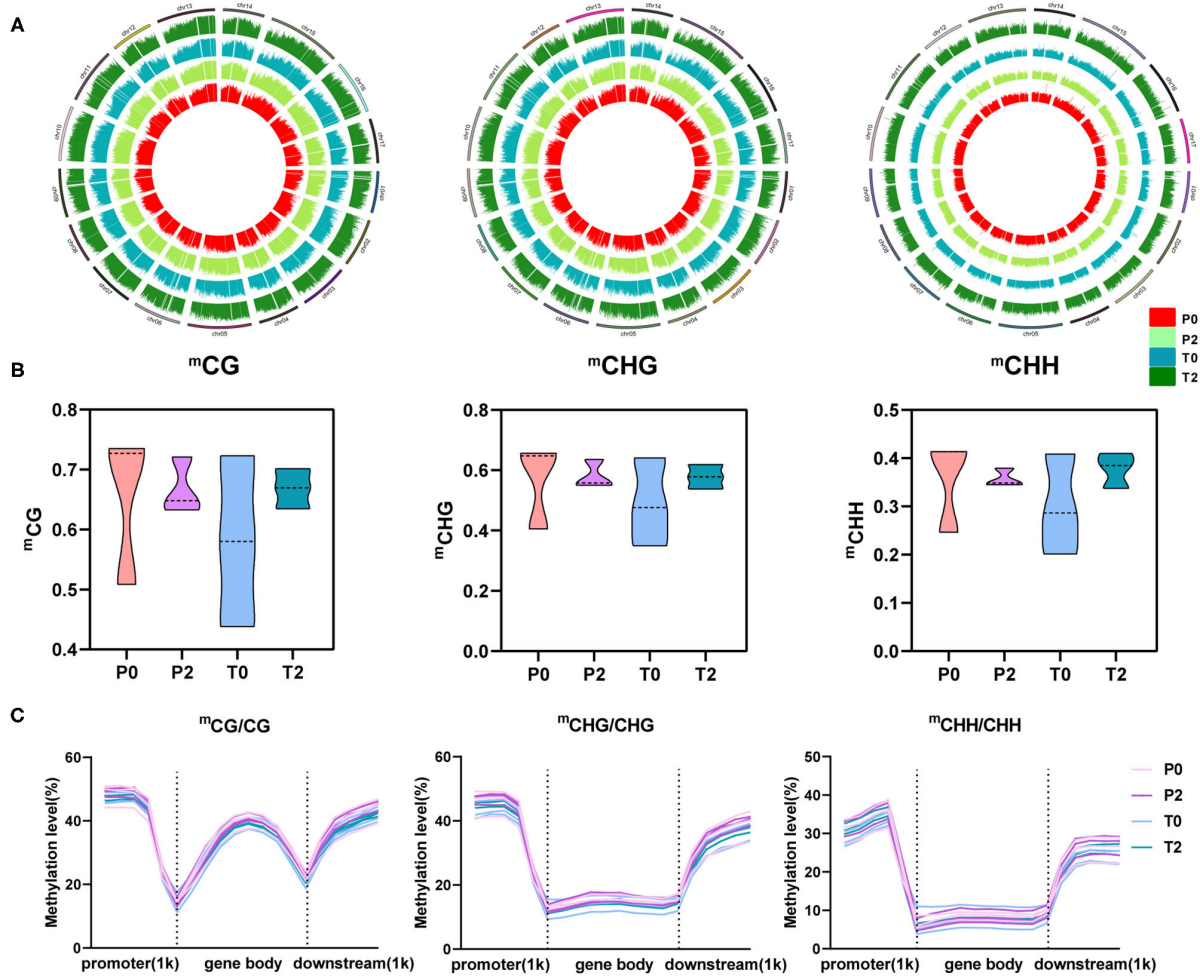
The global profiles of methylation and three types of methylation in “Fuji” apples. **(A)** Methylation profiles on 17 chromosomes. Circle plot of three methylation types on 17 chromosomes in apple. ^m^CG, ^m^CHG, and ^m^CHH from left to right, and P0, P2, T0, and T2 from inside to outside in each circle. **(B)** Mean levels of the three methylation types were plotted on the violin. From left to right are ^m^CG, ^m^CHG, and ^m^CHH. **(C)** Methylation levels among promoter, gene body, and downstream regions and their 1kb regions. From left to right are ^m^CG/CG, ^m^CHG/CHG, and ^m^CHH/CHH.

To obtain more detailed information about methylation during apple browning, we performed single-base whole-genome Bis-sequencing of the P0, P2, T0, and T2 samples and obtained a map of the distribution of methylation across the apple chromosomes. There are three main types of methylation (CG, CHG, and CHH) and CHH has a higher ratio (Figure 2A). We performed a violin chart of the different types of methylation levels, and it is obvious that the methylation levels of the two types of apples are more concentrated in the browning, and the P-type apples have decreased, whereas the T-type apples have increased. But there is no significant difference (*p* > 0.05) between them (Figure 2B). The distribution of different methylation types in the upstream and downstream of the gene can also be seen that the methylation level is the highest in the promoter region, followed by the downstream region, and the gene body region is the lowest (Figure 2C).

### Distribution and Classes of Methylation on Chromosome

**Figure 3:**
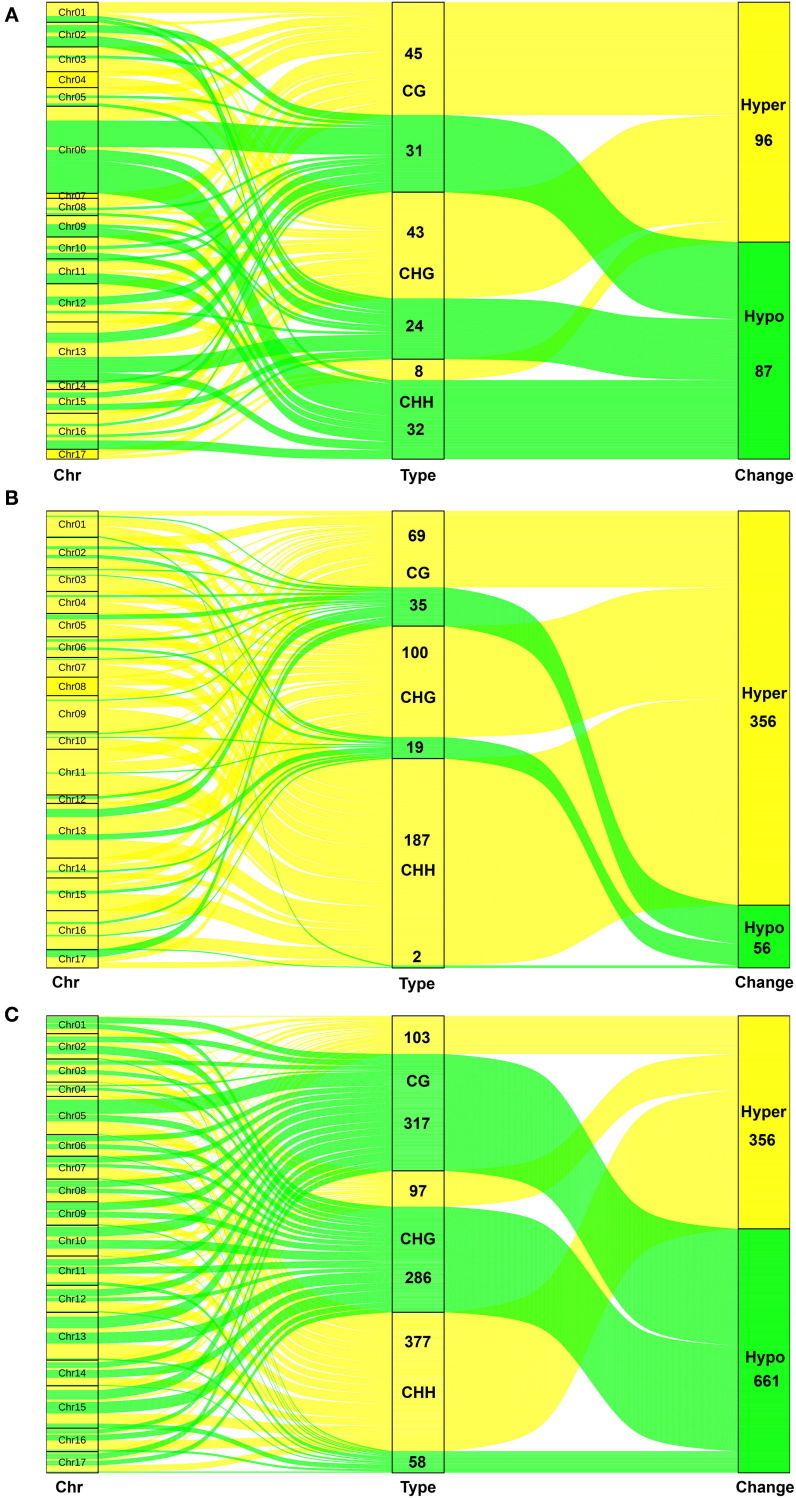
The flow of different methylation types on the 17 chromosomes in three groups. **(A)** The Sankey diagram in P2 vs. P0. **(B)** The Sankey diagram in T2 vs. T0. **(C)** The Sankey diagram in T2 vs. P2. The green lines are hypomethylation (down) and the yellow lines are hypermethylation (up). The left is chromosome (Chr), and the middle is methylation type including CG, CHG, and CHH. The right is change type including hypomethylation (down) and hypermethylation (up).

The methylation classes were displayed by a Sankey diagram (Figure 3). The results showed that the hypermethylation has 96 and the hypomethylation has 87 after the browning of P-type apples (P2/P0). The P2/P0 comparison indicated that the greatest extent of browning-related hypomethylation occurred on Chr06, whereas the hypermethylation was most obvious on Chr04 and Chr17. The proportion of hypermethylation is more than 86% after browning in T-type apples. Therefore, there was a significant hypermethylation after browning in the T-type apples. Hypomethylation mainly occurred on CG and CHG. There are obvious enhancements throughout the chromosomes. But for the comparison of T2/P2, we found that there is little difference between hyper- and hypomethylation. The increase is mainly reflected in CHH, which is distributed on each chromosome.

### DEGs Analysis by Transcriptome

**Figure 4:**
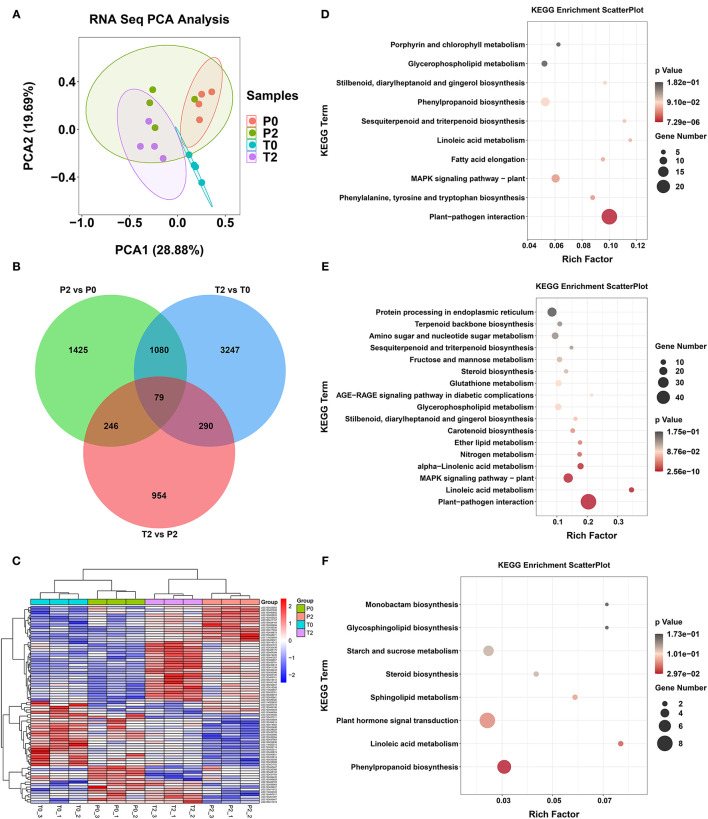
The results in transcriptome. **(A)** PCA of RNA-seq. **(B)** Venn diagram of the transcriptome analysis. **(C)** Heatmap of DEGs from RNA-seq. **(D–F)** KEGG of DEGs from RNA-seq in P2 vs. P0, T2 vs. T0, and T2 vs. P2, respectively.

We profiled the transcriptomics of the P0, P2, T0, and T2 samples using RNA-sequencing. The PCA of RNA- sequencing is shown in Figure 4A. After identifying the DEGs (*p* < 0.05), we found that there were 79 common genes with significant differences among the three comparisons (P2/P0, T2/T0, and T2/P2, Figure 4B). The longitudinal clustering is the samples, and the clustering of samples in the 4 groups is very well by heatmap and cluster analysis on these 79 genes (Figure 4C). The horizontal clustering is the genes, and changes in genes are mainly divided into two categories, one is the genes that increase significantly, and the other is the genes that decrease significantly after browning. Finally, KEGG enrichment analysis was performed on the three comparative differential genes (Figures 4D–F) and found that after browning, whether it is P-type or T-type apple, plant–pathogen interaction is the most significant pathway to increase, and linoleic acid metabolism is significantly increased (Figures 4D,E).

### Different Proteins by Proteomic Analysis

**Figure 5:**
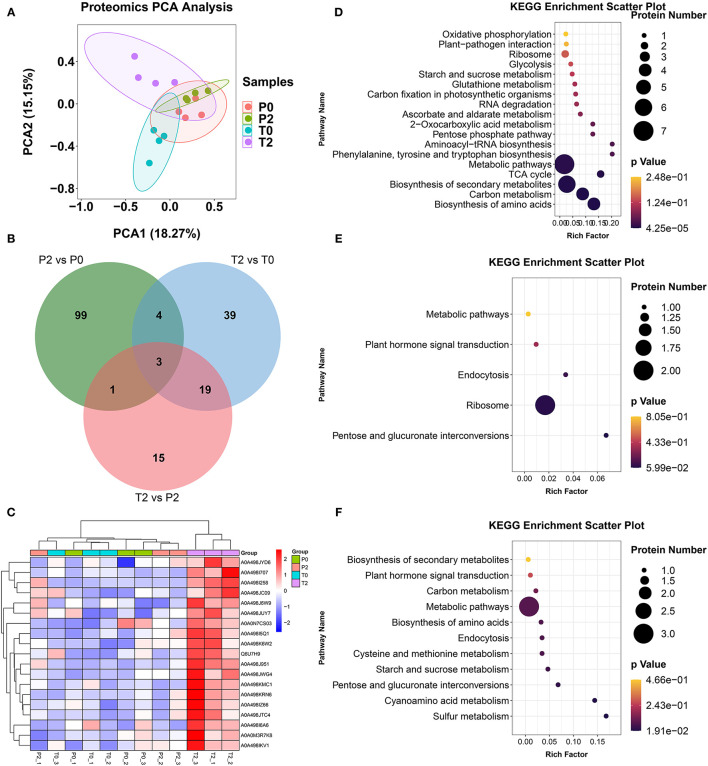
The results in proteomics. **(A)** PCA of proteomics. **(B)** Venn diagram of the proteomics analysis. **(C)** Heatmap of differentially expressed proteins from proteomics. **(D–F)** KEGG of differentially expressed proteins from proteomics in P2 vs. P0, T2 vs. T0, and T2 vs. P2, respectively.

Proteomic analysis obtained a total of 1,976 proteins in 12 samples through label-free proteomic technology. PCA results showed that P2 and T2 had significant differences, indicating that the two types of apples had significant protein differences after browning (Figure 5A). Venn analysis was performed on three comparisons and three proteins are OMT1, transketolase, and Glyco_hydro_18 (Figure 5B). OMT1 is methyltransferase and significantly increased after browning in T-type apples (*p* < 0.05). The heatmap is key different proteins in the four groups, and T2 had a significantly increased (Figure 5C). KEGG enrichment analysis revealed significant changes in metabolic pathways such as biosynthesis of amino acids, carbon metabolism, biosynthesis of secondary metabolism, and the TCA cycle, in P2/P0 (Figure 5D). In T2/T0, the significantly altered metabolic pathways were pentose and glucuronate interconversion (Figure 5E); in T2/P2, the significantly altered metabolic pathways were sulfur metabolism and cyanoamino acid metabolism (Figure 5F).

### Multi-Omics Combined Analysis and Landscape of Apple Browning

**Figure 6:**
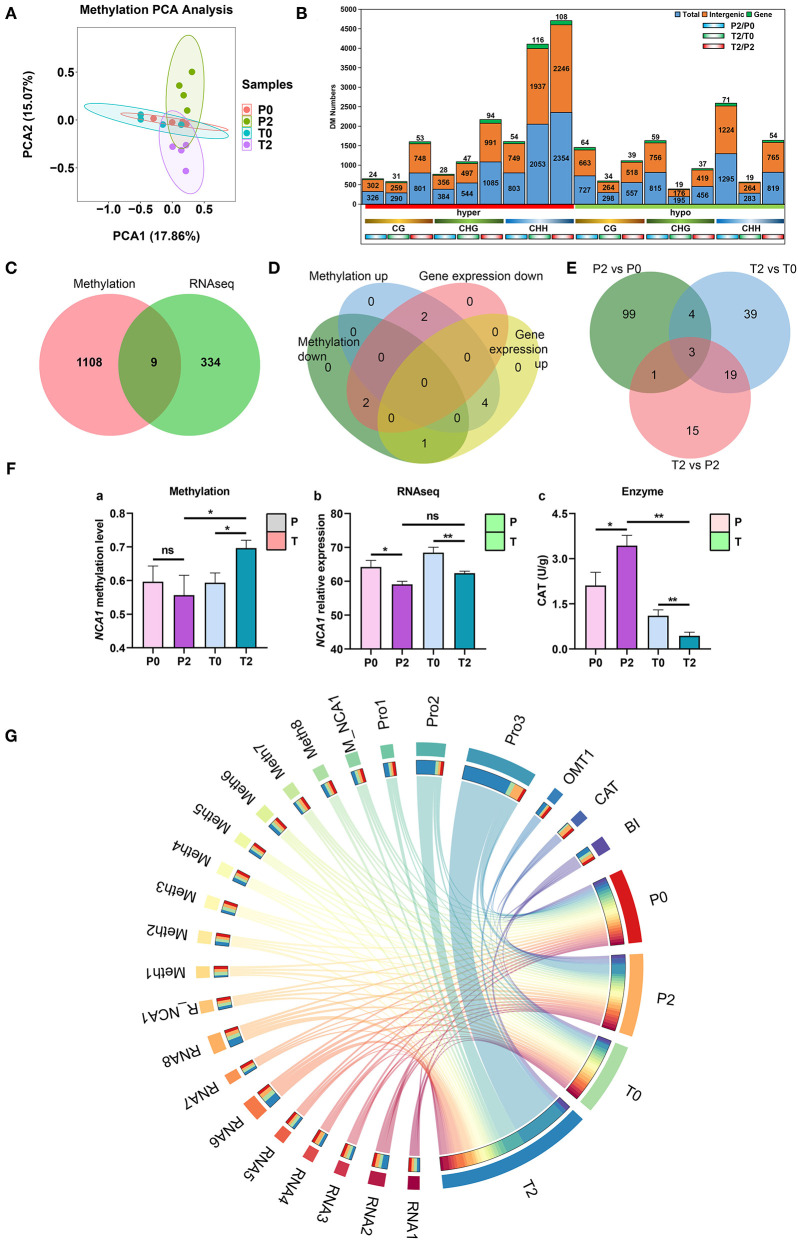
Multi-omics analysis results and browning landscape in “Fuji” apples. **(A)** PCA of all methylation data. Four groups are P0 (red), P2 (green), T0 (blue), and T2 (purple). **(B)** Differential methylation numbers. The number of all DMRs is displayed. In the histogram, the blue ones are all DMRs, the orange ones are intergenic regions, and the green ones are independent genes. Below the horizontal axis, the red on the left is hypermethylation, and the green on the right is hypomethylation. Below, different colors are used to indicate different types of methylation, namely CG, CHG, and CHH. The bottom is three comparisons, from left to right are P2/P0, T2/T0, and T2/P2. Differential methylation regions are *p* < 0.05, fold change > 2. **(C)** The Venn analysis of methylation and RNA-seq. There are nine common differential genes. **(D)** Venn diagram for up- and downregulated methylation genes and DEGs from RNA-seq. Two genes (*NCA1* and *HOS1*) are the upregulation of methylation and the downregulation of gene expression. One gene (*IDD7*) is the downregulation of methylation and the upregulation of gene expression. **(E)** Venn diagram from the proteomics analysis. Three proteins (OMT1, transketolase, and Glyco_hydro_18) with the same differential accumulation trend in the P2/P0, T2/T0, and T2/P2 comparisons. **(F)** The methylation level and expression of *NCA1* gene, and CAT activity (*p* < 0.05). **(G)** Circos diagram in “Fuji” apples browning. The red, orange, green, and blue on the right represent P0, P2, T0, T2, and T2, respectively. The different colors on the left represent the different indexes of four groups. The data of each proportion are shown in Supplementary Table S3. In the figures, ns indicates no significance, *p < 0.05, **p < 0.01.

The multi-omics analysis provides an opportunity to fully and clearly understand the browning process. Here, we employed transcriptomics and proteomics to discover the information of gene and protein expression affected by methylation, which provides clear landscape for the understanding of browning. PCA was performed on all methylomic data (Figure 6A) and divided them into different methylation types for PCA (Supplementary Figure S3). This analysis indicated little difference in terms of methylation between the two types of apples prior to any browning, but there were obvious methylation differences after browning. We then assessed DMRs, and one notable trend was that the total number of methylated regions is mainly in the CHH type and increases after browning (Figure 6B). The highest ones are mainly the difference between T2 and T0, and the difference between T2 and P2. KEGG analysis was performed on the differentially methylated genes (*p* < 0.05) of P2 and T2 with significant differences (Supplementary Figure S4), and it was found that there were significant changes in genes in a variety of metabolic pathways, protein export, phagosome, biosynthesis of secondary metabolites, carbon metabolism, and fatty acid metabolism (*p* < 0.01). The Venn analysis of the differential genes in methylation and transcriptome (T2/T0 and T2/P2 have common different genes) found that there are 9 genes that have differences in both methylation and gene expression (Figure 6C). According to the up- and downregulation of these 9 genes, Venn analysis was carried out, and it was found that *NCA1* and *HOS1* genes had methylation upregulation and expression downregulation (*NCA1* and *HOS1*), and IDD7 is opposite (Figure 6D). Venn diagram from the proteomics analysis showed that three proteins (OMT1, transketolase, and Glyco_hydro_18) with the same differential accumulation trend in the P2/P0, T2/T0, and T2/P2 comparisons (Figure 6E). CAT activity analysis showed that it increased in P-type and decreased in T-type apples, and significantly higher in the P-type than T-type apples (Figure 6F, enzyme). The methylation level of *NCA1* increased significantly and the gene expression decreased significantly (Figure 6F), which may inhibit the activity of CAT (*p* < 0.05). Therefore, we summarized the different results and drew a Circos diagram in apple browning (Figure 6G). When the initial browning occurred, the damage caused by cutting to cells led to a rapid expression of enzymes and genes, especially methylation-related genes. The rapid increase in methylated genes *MdCMT3* and *MdCMT3c* was due to the increase in gene methylation level leading to a significant increase in DNA methylation (5 mC%). *NCA1* was methylated by OMT1 that provided more methylation catalytic activity resulting in the decrease in the expression level of *NCA1* gene. The decline of *NCA1* leads to inhibition of CAT activity, which accelerated apple browning. Multi-data changed significantly in the T2 group, and the highest proportion is displayed in the Circos diagram, which also had the highest BI (Figure 6G, Supplementary Table S3).

## Discussion

Apple browning is complex reaction and has a strong relationship with PPO. In our previous study, a new browning way of non-different PPO was discovered in P-type and T-type “Fuji” apples from natural bud mutations. Additionally, the two types of apples had the same growth environment and genetic background, and this browning difference might be related to DNA methylation in epigenetics. 5′-Aza can delay the non-different PPO browning. Therefore, the difference may be related to epigenetics, such as DNA methylation. The relationships of the changes in genes and proteins of apple during browning are analyzed by multi-omics including methylomics, transcriptomics, and proteomics obtained.

Previous studies found that fruits' DNA demethylase genes were significantly decreased and underwent DNA hypermethylation process in orange fruit ripening (25). The methylation of histidine–lysine demethylase H3K27 encoded by *SlJMJ6* gene to activate the expression of ripening-related genes and regulate fruit ripening, and *SlJMJ6* accelerated the fruit maturation of tomato by the upregulation of a large number of maturation-related genes (48). DNA methylation might be involved in the parent-of-origin effects and affected tomato fruit quality (49). As a result, browning is a process after fruit ripening, and DNA methylation is closely related to fruit maturation.

The level of DNA methylation is determined by methylation and demethylation transferases in browning apples. Maintenance of DNA methylation depends on the cytosine-rich region and is catalyzed by DNA methyltransferases in plants. The METHYLTRANSFERASE 1 (MET1) is an ortholog of the mammalian DNA (cytosine-5)-methyltransferase 1 (DNMT1) and affects DNA replication and methylates (13, 50). We found that the expression level of *MdMET1* was significantly increased in T-type apples compared to P-type apples, suggesting that DNA methylation was likely elevated in T-type apples. The DNA methyltransferase DOMAINS REARRANGED METHYLASE 2 (DRM2) and DNA methyltransferase DOMAINS REARRANGED METHYLASE 3 (DRM3) enzymes catalyze *de novo* DNA methylation in a sequence-independent manner (51). DRM2 maintains methylation through RNA-directed DNA methylation or by CMT2 (52). In our results, *MdDMR2* had a significant downregulation in P-type and T-type apples, but *MdDMR3* had a significant upregulation in T-type apple after browning. DNA methylation in a symmetric CG context is maintained by *MET1*, whereas CHG methylation is maintained by CHROMOMETHYLASE 3 (CMT3) or CMT2 (53, 54). CMT3c is a CMT-type cytosine DNA methyltransferase 3c. In our experiments, *MdCMT2, MdCMT3*, and *MdCMT3c* genes were significantly upregulated in P-type and T-type apples, which suggests a likely increase in CHG and CHH methylation in both types. DNA methylation can be removed by active demethylases. REPRESSOR OF SILENCING 1 (ROS1) is a 5-methylcytosine DNA glycosylase/lyase that inhibits homology-dependent transcriptional gene silencing by demethylating (55). DEMETER (DME) mediated active DNA demethylation and by downregulation of the DECREASED DNA METHYLATION 1 (DDM1) in the *Arabidopsis thaliana* (56, 57). Our results indicated that *MdROS1* and *MdDME* were significantly upregulated in P-type apples after browning.

The catalytic process of methylation and methylated genes may play an important role in the non-different PPP browning. The upregulated OMT1 can provide more methyl substrates to support DNA methylation. OMT1 is required for the production of methylated phenylpropenes in apples (58). We detected that increased accumulation of OMT1 proteins was significantly upregulated, especially in T-type apples. *NCA1* gene has increased methylation levels and decreased expression in the process of browning. *NCA1* is a molecular chaperone of the CAT, which can maintain the activity of CAT. When *NCA1* is inhibited, CAT activity decreases (36). We found that CAT activity significantly decreased after browning in T-type apples, but P-type apples had higher CAT activity, and there was no significant decline in P-type apples.

In conclusion, our data support that upregulated methylation led to *NCA1* hypermethylation and inhibited *NCA1* gene expression. The high expression of OMT1 provides more methyl substrates to significantly increase the methylation level, which inhibited CAT activity and led to more quickly browning. In the future, we will combine metabolomics to uncover the pathways and mechanisms of non-different PPO browning to provide better approaches for quality and storage of fresh-cut apples.

## Data Availability Statement

The original contributions presented in the study are included in the article/Supplementary Material, further inquiries can be directed to the corresponding authors.

## Author Contributions

WW, JZ, ZW, and FW conceived and designed the experiments. LW, TT, and WW conducted the experiments. WW and TT analyzed data. WW and JZ wrote the paper. JZ and FW revised the paper. All authors contributed to the article and approved the submitted version.

## Funding

This work was supported by Agricultural Science and Technology Innovation Program (CAAS-ASTIP-2021-IFST-03).

## Conflict of Interest

The authors declare that the research was conducted in the absence of any commercial or financial relationships that could be construed as a potential conflict of interest.

## Publisher's Note

All claims expressed in this article are solely those of the authors and do not necessarily represent those of their affiliated organizations, or those of the publisher, the editors and the reviewers. Any product that may be evaluated in this article, or claim that may be made by its manufacturer, is not guaranteed or endorsed by the publisher.

## Supplementary Material

Supplementary Figure S1 The change of browning after 5′-Aza treatment. **(A)** Photos of browning status of T-type apples treated by 5′-Aza after 2 h. CK 2 h means T-type apples were treated by 10% DMSO. 5′-Aza 2 h means T-type apples were treated by 5′-Aza solution (dissolved in 10% DMSO). **(B)** BI of CK and 5′-Aza in T-type apples. ^***^ indicates *p* < 0.001.

Supplementary Figure S2 The chromatogram of m5C and C by UPLC-MS/MS.

Supplementary Figure S3 The scores of PCA in different types of methylation. **(A–L)** are the scores of PCA of C exon, CG exon, CHG exon, CHH exon, C gene, CG gene, CHG gene, CHH gene, C promoter2k, CG promoter2k, CHG promoter2k, and CHH promoter2k, respectively.

Supplementary Figure S4 The KEGG of methylation genes. **(A–C)** The KEGG of methylation genes in P2 vs. P0, T2 vs. T0, and T2 vs. P2, respectively. The circle is gene number and the color is *p*-value.

Supplementary Table S1 The compound parameters of LC-MS/MS.

Supplementary Table S2 Primer list of methylation-related genes.

Supplementary Table S3 Different data of multi-omics (data is mean value).
