# Supplementary figures and images for: Multi-Omics Landscape of DNA Methylation Regulates Browning in “Fuji” Apple
Source: Front Nutr. 2022 Feb 7;8:800489. doi: 10.3389/fnut.2021.800489 (PMC8859415; doi:10.3389/fnut.2021.800489)

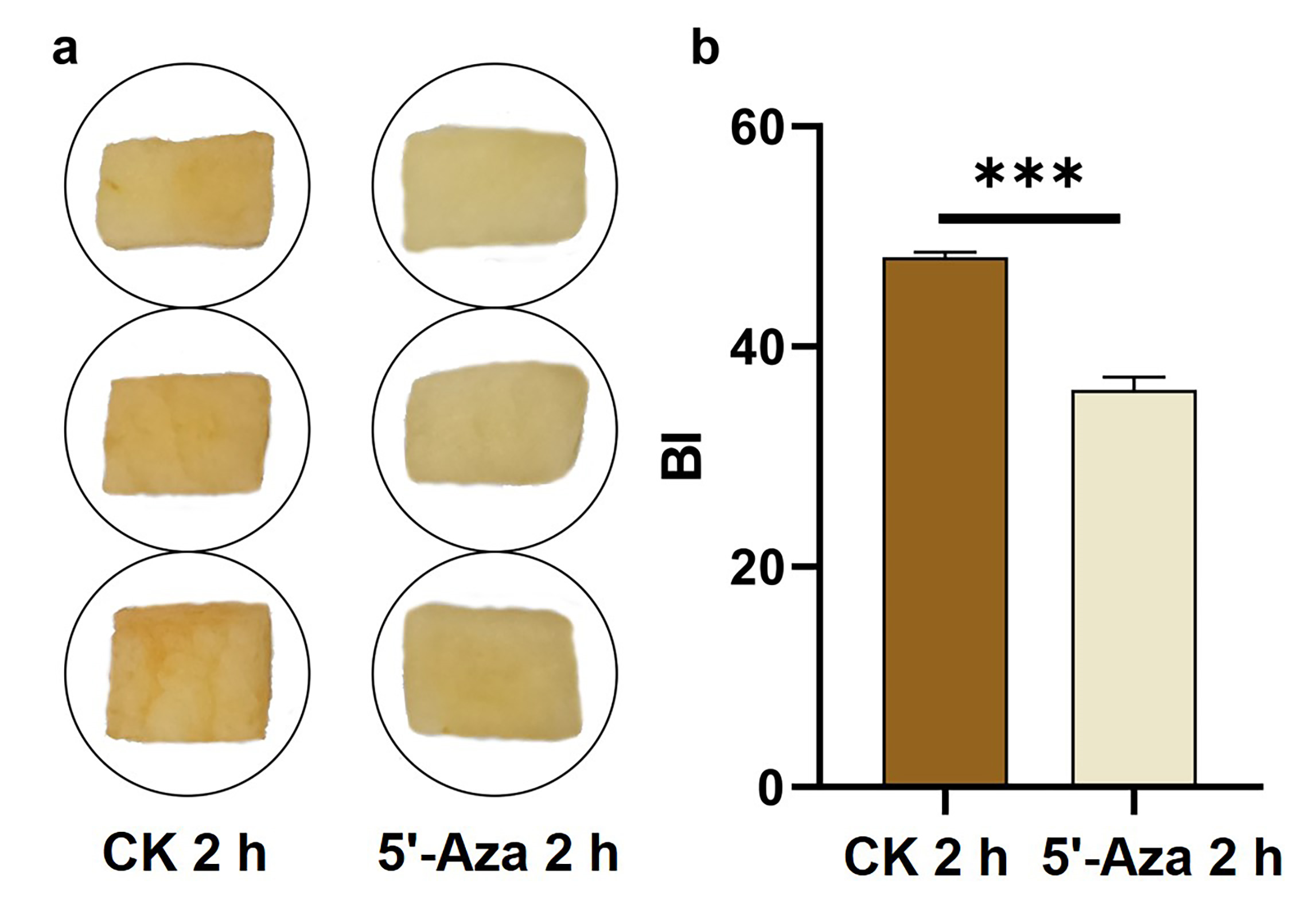

Supplement: Supplementary Figure S1 — The change of browning after 5′-Aza treatment. (A) Photos of browning status of T-type apples treated by 5′-Aza after 2 h. CK 2 h means T-type apples were treated by 10% DMSO. 5′-Aza 2 h means T-type apples were treated by 5′-Aza solution (dissolved in 10% DMSO). (B) BI of CK and 5′-Aza in T-type apples. *** indicates p < 0.001. [file Image_1.TIF]

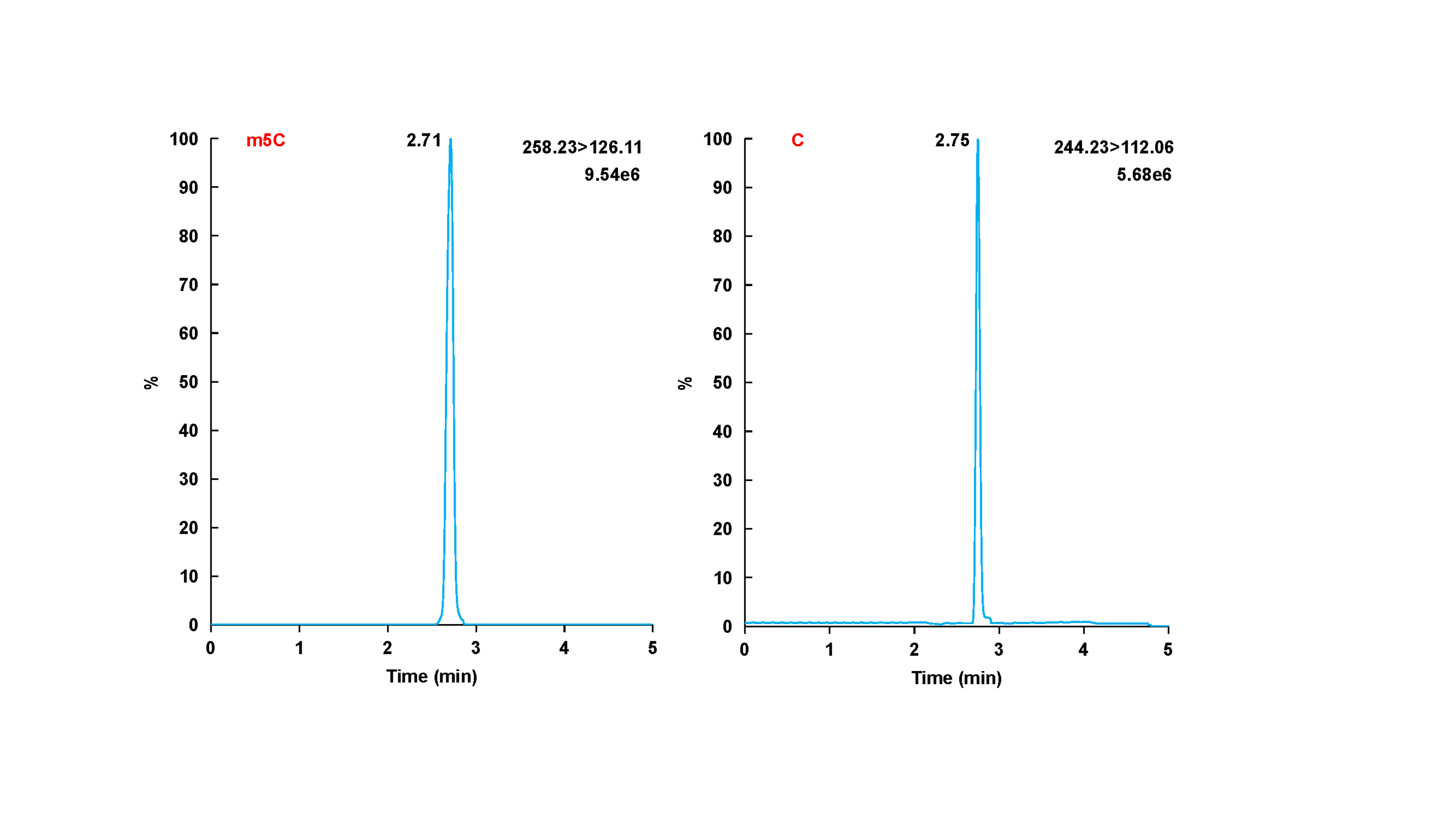

Supplement: Supplementary Figure S2 — The chromatogram of m5C and C by UPLC-MS/MS. [file Image_2.TIF]

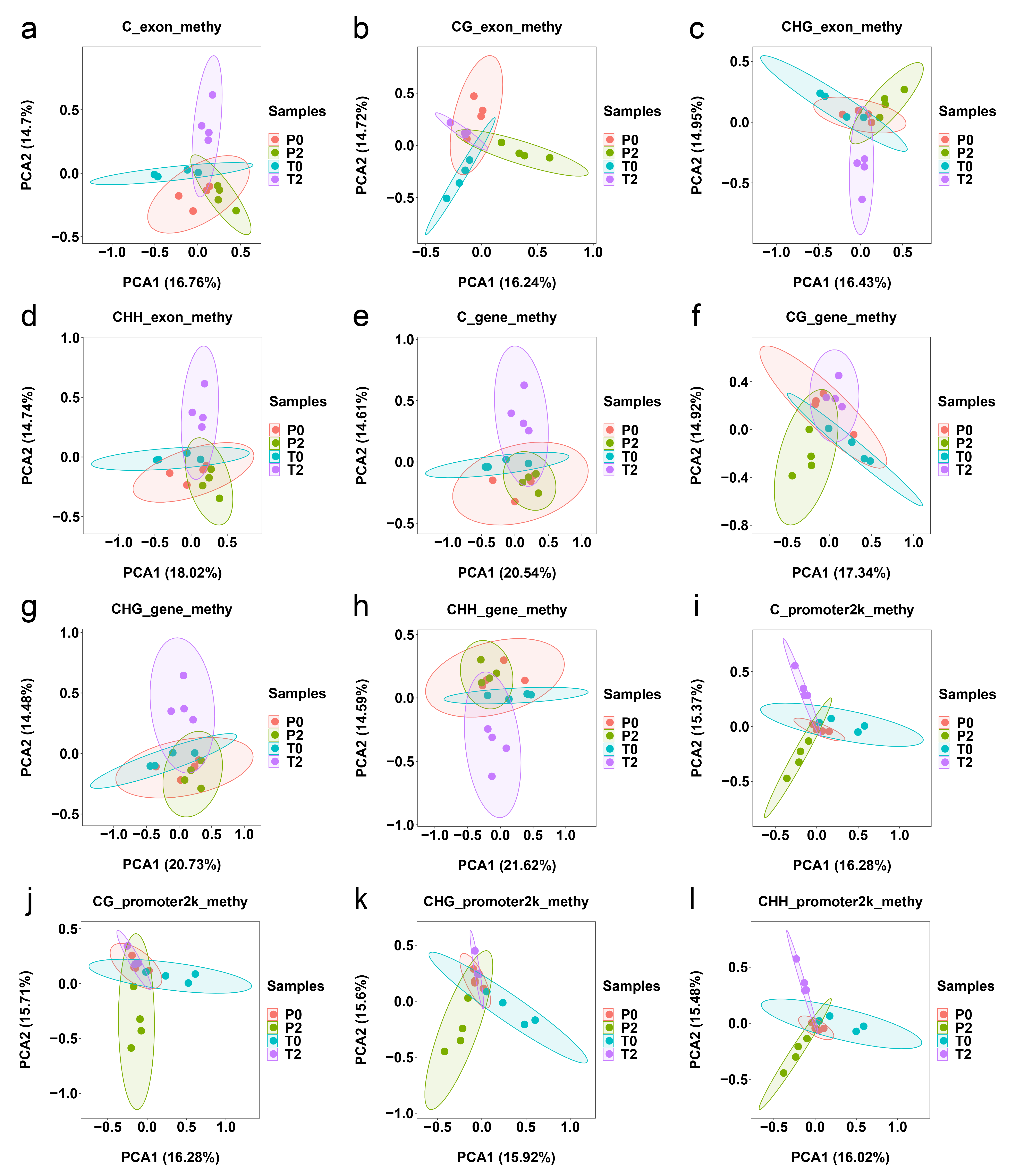

Supplement: Supplementary Figure S3 — The scores of PCA in different types of methylation. (A–L) are the scores of PCA of C exon, CG exon, CHG exon, CHH exon, C gene, CG gene, CHG gene, CHH gene, C promoter2k, CG promoter2k, CHG promoter2k, and CHH promoter2k, respectively. [file Image_3.TIF]

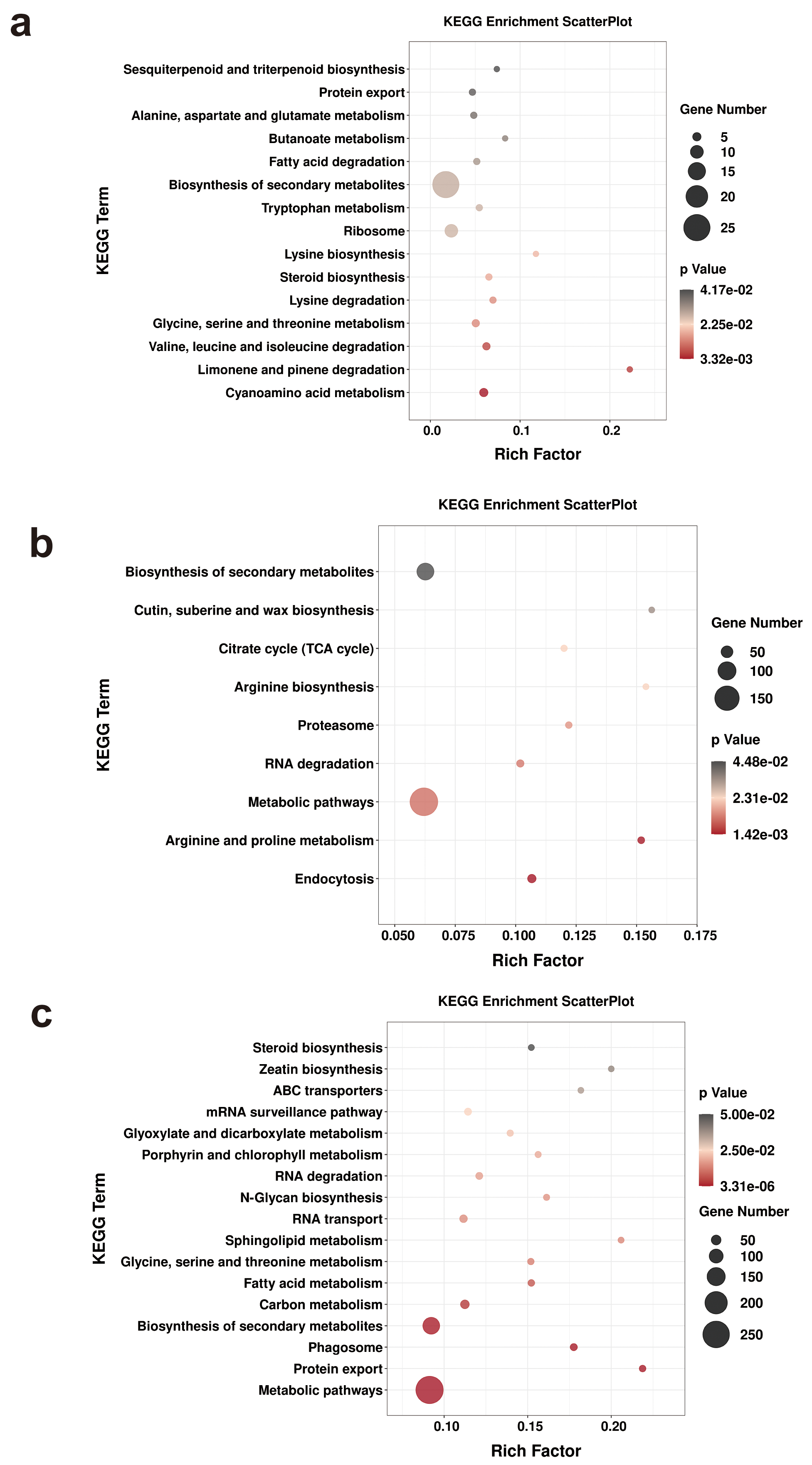

Supplement: Supplementary Figure S4 — The KEGG of methylation genes. (A–C) The KEGG of methylation genes in P2 vs. P0, T2 vs. T0, and T2 vs. P2, respectively. The circle is gene number and the color is p-value. [file Image_4.TIF]
