# Supplementary material for: Multi-Omics Landscape of DNA Methylation Regulates Browning in “Fuji” Apple
Source: Front Nutr. 2022 Feb 7;8:800489. doi: 10.3389/fnut.2021.800489 (PMC8859415; doi:10.3389/fnut.2021.800489)
Supplement: Supplementary Table S1 — The compound parameters of LC-MS/MS. [file Table_1.DOCX]

**Supplementary Table 1. The compound parameters of LC-MS/MS**

| **Compound** | **Parent (m/z)** | **Daughters**  **(m/z)** | **Cone Voltage**  **(V)** | **Collision Energy**  **(V)** | **Ion Mode** | **Comments** |
| --- | --- | --- | --- | --- | --- | --- |
| Cytidine | 244.23 | 112.06 | 28 | 10 | ES+ | quantitative ion pair |
|  | 244.23 | 94.96 | 28 | 46 | ES+ | Qualitative ion pair |
| 5mC | 258.23 | 126.11 | 22 | 10 | ES+ | quantitative ion pair |
|  | 258.23 | 108.89 | 22 | 42 | ES+ | Qualitative ion pair |
| Thymidine (IS) | 243.23 | 127.11 | 16 | 10 | ES+ | quantitative ion pair |
|  | 243.23 | 117.06 | 16 | 6 | ES+ | Qualitative ion pair |

**Supplementary Table 2. Primer list of methylation-related genes**

| **Gene name** | **NCBI ID** | **Forward primer sequence（5'-3'）** | **Reverse primer sequence（5'-3'）** |
| --- | --- | --- | --- |
| *MdMET1* | JX575805.1 | TCCACTGGACAGATAGTTGACTTGA | GGCGCTTGGCTGTGTTG |
| *MdDRM2* | XM_029096698.1 | GGAGCCCGTGCAACAATCTA | CCTCCAGTCCATCACGATGA |
| *MdDRM3* | XM_029091507.1 | CGAGAATAGGTTTCGCATTGTTC | TGTGGCATTGCATCCTCAA |
| *MdCMT2* | XM_029104418.1 | GGAGCAAATTTCCGGGATCTAC | CTCTCTCCGAGCCACATTGTC |
| *MdCMT3* | NM_001293872.1 | AATATTCGCTTGCGGGTTTG | CAGACACGGTGGCCATCAC |
| *MdCMT3c* | JX575804.1 | GGTGCCTATGGGCTTCCA | CGGGCCCCCCACAA |
| *MdDDM* | XM_017322621.2 | GCGGTATGAAAGGAAAGCTTAACA | GGTCAGGATGATTGCAGTTCTTC |
| *MdROS* | XM_008388713.3 | CCACCTGACCAAGCAAAAGAGTA | CACGCTTTTCAACCCCAATC |
| *MdDME* | XM_029108570.1 | CAAGTTAATGAGATGTTCGCAGATC | CACCCCCTTGGAACATCAAT |
| *MdEF-1α* | XM_008387060.3 | TGCTTTCACTCTTGGTGTCA | GGTAGGATGAGACTTCCTTC |

**Supplementary Table 3. Different data of multi-omics (data is mean value)**

| Class | Name | Name abbreviation | P0 | P2 | T0 | T2 |
| --- | --- | --- | --- | --- | --- | --- |
| RNA seq | RNA-LOC103403990 | RNA1 | 1 | 0.88 | 1.05 | 0.97 |
| RNA seq | RNA-LOC103449592 | RNA2 | 1 | 1.23 | 1.05 | 2.08 |
| RNA seq | RNA-LOC103445437 | RNA3 | 1 | 0.99 | 1.04 | 0.89 |
| RNA seq | RNA-LOC103425033 | RNA4 | 1 | 1.40 | 0.28 | 1.02 |
| RNA seq | RNA-LOC103432994 | RNA5 | 1 | 0.81 | 1.02 | 0.92 |
| RNA seq | RNA-LOC103418780 | RNA6 | 1 | 1.55 | 1.56 | 2.60 |
| RNA seq | RNA-LOC103424637 | RNA7 | 1 | 0.75 | 0.79 | 0.89 |
| RNA seq | RNA-LOC103435892 | RNA8 | 1 | 1.35 | 1.32 | 2.41 |
| RNA seq | RNA-NCA1 | R_NCA1 | 1 | 0.92 | 1.07 | 0.97 |
| Methylation | Meth-LOC103403990 | Meth1 | 1 | 0.99 | 1.23 | 0.58 |
| Methylation | Meth-LOC103449592 | Meth2 | 1 | 0.89 | 0.99 | 1.23 |
| Methylation | Meth-LOC103445437 | Meth3 | 1 | 1.10 | 1.16 | 0.89 |
| Methylation | Meth-LOC103425033 | Meth4 | 1 | 1.00 | 0.89 | 1.21 |
| Methylation | Meth-LOC103432994 | Meth5 | 1 | 0.82 | 0.81 | 0.99 |
| Methylation | Meth-LOC103418780 | Meth6 | 1 | 0.96 | 1.01 | 1.20 |
| Methylation | Meth-LOC103424637 | Meth7 | 1 | 1.05 | 1.04 | 0.85 |
| Methylation | Meth-LOC103435892 | Meth8 | 1 | 0.83 | 0.88 | 1.20 |
| Methylation | meth-NCA1 | M_NCA1 | 1 | 0.93 | 1.00 | 1.17 |
| Proteomics | A0A498IEQ7 | Pro1 | 1 | 0.70 | 0.71 | 1.67 |
| Proteomics | A0A498JIB6 | Pro2 | 1 | 0.82 | 1.02 | 6.72 |
| Proteomics | A0A498KPG4 | Pro3 | 1 | 3.35 | 1.60 | 16.89 |
| Proteomics | OMT1 | OMT1 | 1 | 0.62 | 0.33 | 1.40 |
| Enzyme | CAT | CAT | 1 | 1.63 | 0.52 | 0.21 |
| BI | Browning Index | BI | 1 | 1.16 | 1.00 | 1.68 |
